# Additive value to clinical judgement of blood neutrophil gelatinase-associated lipocalin in diagnosis of acute kidney injury and prediction of mortality in patients hospitalized from the emergency department

**DOI:** 10.1186/cc10961

**Published:** 2012-03-20

**Authors:** L Magrini, B De Berardinis, R Marino, G Gagliano, E Ferri, P Moscatelli, P Ballarino, B Gliozzo, G Carpinteri, S Di Somma

**Affiliations:** 1 S. Andrea Hospital 'Sapienza' University, Rome, Italy; 2 S. Martino Hospital, Genoa, Italy; 3 S. Elia Hospital, Catania, Italy

## Introduction

Acute kidney injury (AKI) is a common and difficult to diagnose complication among hospitalized patients with increasing incidence.

## Methods

A total of 665 (357 M:308 F; mean age 74 ± 14 years) emergency department (ED) patients designated for hospitalization were included in a multicenter prospective study to evaluate the utility of blood neutrophil gelatinase-associated lipocalin (NGAL) assessments as an aid in the early risk evaluation for AKI. NGAL and serum creatinine (sCr) were determined at ED presentation (T0), 6, 12, 24 and 72 hours after hospitalization. The clinical certainty of AKI was determined by ED physician (Ph) while blinded to NGAL results.

## Results

Preliminary diagnosis of AKI by the ED Ph occurred in 218/665 patients (33%). Final adjudicated AKI clinical diagnosis was confirmed in 49/665 patients (7%). The AUC for NGAL alone in the final diagnosis of AKI was 0.80 (± 0.07). When NGAL was added to the ED Ph's clinical judgement in a logistic model, the AUC was increased to 0.89 (± 0.06). The AUCs for the additional endpoints are shown in Table 1. When the same model combining NGAL with the ED Ph's clinical judgement was compared to a clinical model combining T0 sCr results with the ED Ph's clinical judgement, the net reclassification index was 32.4%, meaning that the correction classification of AKI improved 32.4 percentage points.

**Table 1:** 

	Event	No event	AUC (95% CI)
Diagnosis of AKI	49	616	0.80 (0.07)
RIFLE	25	640	0.72 (0.12)
sCr bump	10	655	0.85 (0.10)
Oliguria	14	651	0.81 (0.14)
Mortality	27	638	0.76 (0.11)

## Conclusion

Our study demonstrated that blood NGAL measurements in patients hospitalized from the ED for critical conditions may improve the clinical diagnosis of AKI development. The routine use of NGAL in the ED may provide utility in deciding the appropriate treatment and management strategies for patients at risk for AKI development.

